# Clinical features of pulmonary cryptococcosis among patients with different levels of peripheral blood CD4^+^ T lymphocyte counts

**DOI:** 10.1186/s12879-017-2865-z

**Published:** 2017-12-13

**Authors:** Qian He, Yuan Ding, Wei Zhou, Hongxing Li, Ming Zhang, Yi Shi, Xin Su

**Affiliations:** 1 Department of Respiratory and Critical Care Medicine, Southern Medical University, Jinling Hospital, Nanjing, 210002 China; 2 Department of Respiratory and Critical Care Medicine, Jinling Hospital, Medical School of Nanjing University, Nanjing, 210002 China; 3 Department of Respiratory Medicine, Third Affiliated Hospital of Soochow University, Changzhou, China

**Keywords:** Pulmonary cryptococcosis, CD4^+^ T cell, Clinical feature, Chest radiography

## Abstract

**Background:**

The clinical manifestation of pulmonary cryptococcosis varies notably between immunocompromised and immunocompetent patients. To better understand pulmonary cryptococcosis, we compared the clinical features of pulmonary cryptococcosis patients with or without decreased peripheral blood CD4^+^ T cell counts.

**Methods:**

We retrospectively reviewed the medical records of 80 patients with cryptococcosis who had been treated in Jingling Hospital from January 2011 to January 2016. According to the normal range of peripheral blood CD4 + T-lymphocyte counts in our population, we chose CD4 = 378/μL as a cut-off value.

**Results:**

The proportion of fever in the patients with decreased CD4+ T cells was higher than that of the patients with a normal amount of CD4+ T cells (86.7% vs 28.6%, *P* < 0.001). The incidence of clinical symptoms, such as cough (60.6% vs 64.7%, *P* = 0.729), chest pain (9.1% vs 26.5%, *P* = 0.064), and dyspnea (27.3% vs 23.5%, *P* = 0.725) showed no difference between patients with low CD4^+^ T cell counts and those with normal CD4+ T cell counts. The number of asymptomatic patients in the CD4^+^ T cell normal group was higher than that in the decreased CD4^+^ T cell group (17.1% vs 0%, *P* = 0.005). Nodules, masses, and halo signs were more common in the CD4^+^ T cell normal patients than in the low-CD4+ T cell patients (79.4% vs 54.5%, *P* = 0.03). The opposite trend was observed for cavitations (14.7% vs 51.5%, *P* = 0.001). The other CT findings, including pulmonary consolidation (*P* = 0.205), and pleural effusion (*P* = 0.641), did not differ significantly between the two groups.

**Conclusions:**

CD4^+^ T lymphocytes have a significant impact on the clinical and radiological characteristics of pulmonary cryptococcosis. The patients with normal CD4^+^ T cell counts were found to have less fever and more nodule-like radiographic findings.

**Trial registration:**

2011NJKY-023-01. Registered on January 10, 2011.

## Background

Cryptococcosis is a fungal infection that may cause serious pulmonary lesions and meningitis. In 2009, Benjamin et al. found that approximately two thirds of patients died within three months after an infection was diagnosed [1]. Additional predisposing factors included underlying solid organ transplantation [2], aggressive cancer treatment, use of immunosuppressants or glucocorticoids, connective tissue diseases, or conditions that may damage immune function [3]. At the same time, an increasing numbers of reports have found that cryptococcosis may happen in immunocompetent patients [4–6]. According to those reports, clinical manifestation can differ considerably between immunocompromised and immunocompetent patients.

CD4+ T lymphocytes play a pivotal role in protecting the body from *Cryptococcus* infection. The CD4+ T-cell immune response may be developed through *Cryptococcus* mannosylation [7], which can recruit macrophages and granulocytes to the lungs in pulmonary cryptococcosis [8]. In a cryptococcal meningitis mouse model, CD4+ T cells were also found to mediate fungal clearance [8]. A clinical study found that the use of rabbit anti-thymocyte globulin (ATG) or alemtuzumab is associated with increased cumulative incidence of cryptococcosis in organ transplant recipients. These medicines can induce substantial decreases in CD4 + T cells [8]. CD4 + T-lymphocyte deficiency is one of a main predisposing factors of cryptococcosis, whereby a CD4+ T-cell count below 100 cells/μl and detectable serum cryptococcal antigen portend high risk for HIV-associated cryptococcosis [9, 10]. Cryptococcosis has also been observed in idiopathic CD4 lymphocytopenia [11, 12]. In our previous study, we determined that low peripheral blood CD4+ T cells may cause more dissemination [13]. In previous studies, patient grouping based on immune status was mainly based on underlying diseases and suffered from a lack of objective criteria [6, 14]. In this study, we analyzed the clinical features, chest images, and prognosis of pulmonary cryptococcosis in patients with different peripheral blood CD4+ T lymphocyte counts.

## Methods

This retrospective study was carried out in Nanjing Jinling Hospital. Records of patients with definite or probable cryptococcosis who were admitted during a five-year period (from January 2011–January 2016) were examined. Patient demographics, underlying disease, clinical manifestations, computed tomography (CT), diagnosis, treatment, and prognosis were analyzed. The relevant follow-up data were obtained through regular clinical interviews or via telephone calls. The last follow-up information was collected on May 30, 2016.

### Diagnosis of cryptococcosis

A definite diagnosis of cryptococcosis was made if the patient met at least one of the following conditions: (1) positive histopathology from tissue samples acquired by open lung biopsy, percutaneous lung biopsy, transbronchial biopsy, or skin biopsy; or (2) positive culture of *Cryptococcus* from cerebrospinal fluid (CSF) or blood. Probable diagnostic criteria were: patients with a positive cryptococcal capsular polysaccharide antigen test in CSF or serum and patients who presented typical clinical manifestations [15, 16]. Exclusion criterion: the patient had no CD4+ T-lymphocyte count when diagnosed with cryptococcosis.

We used flow cytometry to determine the CD4 + T-lymphocyte count in patients’ peripheral blood. The normal range of CD4 + T-lymphocyte counts varied depending on the population. According to the standard used in our laboratory, the normal CD4 + T-cell value was 691/μL ± 273/μL (95% reference range:378/μL–1085/μL). For this reason, we chose CD4+ T cells of less than 378/μL as a cut-off value. The APACHE II score was used to evaluate the disease severity, which was determined according to the patient’s condition prior to antifungal therapy.

### Statistical analysis

The chi-square test was used for inter-group comparisons with categorical variables. Continuous variables were analyzed by Independent Samples t test. All of the data were analyzed with SPSS version 20.0 for Windows. *P* values < 0.05 were considered to be statistically significant.

## Results

### Patient demographics

We examined the records of 80 patients who were diagnosed with cryptococcosis from January 2011 to January 2016. Among these cases, the CD4+ T-lymphocyte counts were equal to or less than 378/μL in 45 patients and the others patients were higher than 378/μL. There were no significant differences between the two groups with respect to gender (*P*>0.05, Table 1). The underlying diseases of the two groups are summarized in Table 1. In the normal CD4+ T cell group, 53.3% (24/45) of patients had used immunosuppressants or corticosteroids. Likewise, 28.6% (10/35) of patients in the low CD4+ T cell group had used immunosuppressants or corticosteroids (Table 1).

**Table 1:** Demographic features and underlying diseases of 80 patients with cryptococcosis

	CD4 ≤ 378/μL *N* = 45 (%)	CD4 > 378/μL *N* = 35 (%)	*P*-value
Male (N)	28 (62.2)	25 (71.4)	0.39
Age (years)			
Mean ± SD	40.3 ± 14.0	44.2 ± 13.2	0.210
Range	16–78	18–71	
Underlying disease			
AIDS	14 (31.1)	0	
SOT	9 (20)	3 (8.6)	
Autoimmune disease	11 (24.4)	2 (5.7)	
Renal disease	3 (6.7)	4 (11.4)	
Blood disease	1 (2.2)	1 (2.9)	
Diabetes mellitus	1 (2.2)	4 (11.4)	
Malignant tumor	2 (4.4)	0	
Other diseases	1 (2.2)	4 (11.4)	
No diseases	3 (6.7)	17 (48.6)	<0.001
Medication history			
Corticosteroids	24 (53.3)	10 (28.6)	0.026
immunosuppressants	18 (40)	6 (17.1)	0.027
Exposure history	3 (6.7)	3 (8.6)	

*AIDS* acquired immune deficiency syndrome, *SOT* solid organ transplant, Exposure history: close contact with animal excreta, such as pigeon droppings or other natural materials contaminated by fungi

### Clinical features

Six patients in the normal CD4+ T cell group (6/35,17.1%) had no symptoms but they were admitted due to the detection of radiographic shadows during chest X-rays at a check-up. All of the participants in the low CD4+ T cell group had symptoms, and the most common symptoms were fever (39/45,86.7%), which was only seen in 10 patients in the normal CD4+ T cell group (10/35,28.6%, *P* < 0.001). There was no difference between the two groups in the incidence of clinical symptoms, such as cough (20/33 vs 22/34, *P* = 0.729), chest pain (3/33 vs 9/34, *P* = 0.064), and dyspnea (9/33 vs 8/34, *P* = 0.725). Differences between the two groups in the prevalence of headache, dizziness (*P* = 1.0) and meningeal irritation (*P* = 0.624) did not reach statistical significance. Concerning the APACHE II score, there was a significant difference between the two groups (*P* < 0.001) (Table 2).

**Table 2:** Clinical characteristics of 80 patients with cryptococcosis

	CD4 ≤ 378/μL *N* = 45 (%)	CD4 > 378/μL *N* = 35 (%)	*P* value
Clinical characteristics			
Asymptomatic	0	6 (17.1)	0.005
Fever	39 (86.7)	10 (28.6)	<0.001
Pulmonary symptoms	33 (73.3)	34 (97.1)	
Cough and expectoration	20 (60.6)^a^	22 (64.7)^a^	0.729
Chest pain	3 (9.1)^a^	9 (26.5)^a^	0.064
Dyspnea	9 (27.3)^a^	8 (23.5)^a^	0.725
Neurological symptoms	34 (75.6)	4 (11.4)	
Headache, dizzy	30 (88.2)^b^	4 (100)^b^	1.000
Meningeal irritation	19 (55.9)^b^	3 (75)^b^	0.624
APACHE II score	9.1 ± 3.7	3.2 ± 4.7	<0.001

^a^The proportion of patients with pulmonary involvement

^b^The proportion of patients with brain involvement

### Chest radiography

The CT scan data were collected from 67 patients, including 33 patients in the low CD4+ T cell group and 34 patients in the normal CD4+ T cell group. The characteristics of the images are given in Table 3. Of the 67 patients, most patients’ pulmonary lesions were within a single lobe. There was no statistically significant difference between the low CD4+ T cell group and the normal CD4+ T cell group when we compared the proportion of single lobe lesions(19/33 vs 18/34,*P* = 0.703). The distribution of lesions was predominately peripheral and adjacent to the pleura. The difference between the two groups did not reach statistical significance (30/34 vs 28/33, *P* = 0.962). The difference in proportion of pulmonary consolidation between the two groups was not significantly different (*P* = 0.205). Nodules/masses were more common in the CD4+ T cell normal group than that in the CD4+ T cell decreased ones.(27/34 vs 18/33, *P* = 0.03). Cavitations within the nodules/masses were more frequent in the CD4+ T cell decreased patients (17/33 vs 5/34, *P* = 0.001). The halo sign was significantly less frequent in the low CD4+ T cell patients than in the normal CD4+ T cell patients (6/33 vs 15/34, *P* = 0.022). Pleural effusion was rare in both of the groups (4/33 vs 2/34, *P* = 0.641) (Table 3).

**Table 3:** Radiological patterns of pulmonary abnormalities of CT scans

	CD4 ≤ 378/μL *N* = 33 (%)	CD4 > 378/μL *N* = 34 (%)	*P* value
Distribution of lesions			
Solitary (single lung)	4 (12.1)	7 (20.6)	0.173
Multiple (single lung)	15 (45.5)	11 (32.4)	0.271
Multiple (bilateral lung)	14 (42.4)	16 (47.1)	0.703
Adjacent to the pleura	28 (84.8)	30 (88.2)	0.962
Lesion characterization			
Nodule or mass	18 (54.5)	27 (79.4)	0.030
Consolidations	10 (30.3)	15 (44.1)	0.205
Mixed alterations	4 (12.1)	8 (23.5)	0.223
Cavitations	17 (51.5)	5 (14.7)	0.001
Halo sign	6 (18.2)	15 (44.1)	0.022
Pleural effusion	4 (12.1)	2 (5.9)	0.641

### Treatment

Treatments included surgery, medication, and a combination of both. In the CD4^+^ T cell decreased group, all of the patients received antifungal therapy. In the normal CD4+ T cell group, two patients were treated with surgery alone, two had surgery and postsurgical medication, and 31 received medication alone.

### Prognosis

The outcome was 90-day all-cause mortality. Mortality attributable to cryptococcosis was also determined. In the low CD4+ T cell group, 13 patients died, most of whom (11 patients) had serious cryptococcal meningitis. In the normal CD4+ T cell group, only two patients died of cryptococcal meningitis. During the follow-up, no relapse was observed in any of the four cases that received pulmonary surgical treatment. The majority of the patients who completed drug therapy in both groups are still alive and the lesions had shrunk significantly and did not subsequently grow (Table 4).

**Table 4:** Treatment and prognosis of 80 patients with cryptococcosis

	CD4 ≤ 378/μL *N* = 45 (%)	CD4 > 378/μL *N* = 35 (%)	*P* value
Prognosis			
Survival	32 (71.1)	33 (94.3)	0.008
Death	13 (28.9)	2 (5.7)	

## Discussion

Cell immunity, especially the CD4 + T-cell-mediated immune response, plays important roles in protection against *Cryptococcus* infection. Currently, the related data of CD4 + T cells concerning cryptococcosis were from small sample studies or case reports [11]. Articles related to the assessment of the severity of cryptococcosis and prognosis through CD4 + T cells are rare. For this reason, the purpose of this study was to retrospectively characterize the clinical features, radiological characteristics, and outcomes of patients with different CD4+ T-lymphocyte counts.

In general, males are more frequently infected than females [17]. In the current study, the disease was also predominant in males. In addition to HIV and organ transplants, connective tissue disease (CTD), chronic leukemia, tumors, and diabetes have also been shown to predispose patients to cryptococcosis [18]. According to the classification method used in previous studies, patients with no underlying disease or diabetes mellitus patients were classified as the immunocompetent group. However, in the current study, we found that some of the patients had lower CD4 + T-lymphocyte counts. While these participants shared many of the same basic diseases as transplant patients, they tended to have lower CD4 + T-lymphocyte counts, but there were still some patients with normal CD4 + T counts. For this reason, the CD4 + T-lymphocyte counts may objectively represent the patient’s immune status.

In addition to host immune factors, cryptococcosis can also be related to environmental exposure to contaminated airspace, including close contact with animal excreta, especially pigeon droppings or other natural materials contaminated by fungi [19]. In this study, only six patients had a history of direct exposure to pigeon droppings, while in other studies, nearly half of the cryptococcosis patients had direct or potential environmental exposure history in south China [20]. This may be because the patients’ exposure history records are not comprehensive. These data suggest that we should pay more attention to the details of potential exposure risk factors of fungal spores.

In recent studies, nearly one third of immunocompetent patients with cryptococcosis were asymptomatic [21]. In the current study, 17.1% of patients with normal CD4+ T cell counts and cryptococcosis were asymptomatic, and their disease was detected incidentally during routine chest radiography or follow-up for other diseases. This situation can also be manifested in other common diseases of the respiratory system, including lung cancer. In this way, pulmonary cryptococcosis is likely to be misdiagnosed.

The most common symptom of cryptococcosis in the current study was fever (61.3%). Some studies have shown that HIV-infected patients are more likely to be febrile than non-immunosuppressed patients [22]. This study produced similar findings. The proportion of fever in patients with low CD4+ T cell counts (86.7%) was higher than that in patients with normal CD4+ T cell levels (28.6%). We speculate that low CD4+ T cell patients have a greater *Cryptococcus* burden and more brain involvement, which may cause more systemic inflammation and consequently fever symptoms. The other symptoms, such as coughing and expectoration, headache and dizziness, chest pain, and dyspnea showed no significant differences between the two groups.

In the previous study, APACHE II scores of immunosuppressed patients were similar to those of HIV patients and also markedly higher than non-immunosuppressed patients (*P* = 0.002) [22]. In the current study, the APACHE II scores were significantly lower in the patients with low CD4+ T cells than in the CD4+ T cell normal patients (*P* < 0.001). According to the description above (infection site/clinical feature/APACHE II scores), the results showed that the CD4+ T-lymphocyte counts can effectively assess the disease severity of cryptococcosis patients.

The radiographic features of pulmonary cryptococcosis were varied. Previous studies have focused on radiographic findings in immunocompetent cohorts or immunocompromised individuals based on underlying disease [14, 23]. The most common finding of chest imaging of pulmonary cryptococcosis in the current study was nodules or masses (54.5% in CD4+ T cell decreased patients and 79.4% in CD4+ T cell normal patients), but the results showed that the nodules/masses were significantly more common in the patients with normal CD4+ T cell counts than in those with low CD4+ T cell counts. This differs from findings reported by Xie et al., who indicated that there was no statistically significant difference between immunocompetent and immunocompromised patients with respect to underlying disease [14]. Chang et al. found that cavitation within nodules/masses was more common in immunocompromised patients than that in immunocompetent ones (*P* = 0.05), which is similar to the current findings [6]. The halo sign was significantly less frequent in the patients with low CD4+ T cell counts than that in the patients with normal CD4+ T cell counts (18.2% vs 44.1%, *P* = 0.022). However, in another study, Xie et al. reported that there was not a significant difference between the two groups according to underlying diseases [14]. Other less common findings, such as pleural effusion, which was also rare in our study [14](12.1% in the patients with low CD4+ T cell counts and 5.9% in the patients with normal CD4+ T cell counts, *P* = 0.64).

If cryptococcal spores reach the lung, they are predisposed to deposition in the peripheral subpleural alveoli [24]. In the current study, lesions were peripherally located in 86.6% of the 67 patients (84.8% in the patients with low CD4+ T cell counts and 88.2% in the patients with normal CD4+ T cell counts, *P* = 0.962), which is similar to the findings of the studies published by Lacomis et al. [25] and Fox et al. [26] (66.2% and 80%, respectively). We also found the lesions to be distributed mainly across single lung. This performance may be associated with the manner in which the spores were inhaled.

Pathologically, most of the nodules and masses were characterized by a granulomatous reaction. The response was composed of macrophages with epithelioid features, multinucleated giant cells, lymphocytes, and langhans type [27]. A Japanese study found that complete granuloma formed with central fibrosis in immunocompetent groups [28]. In the current study, this pathologic reaction was prone to primarily occur in individuals with normal CD4+ T cell counts because these patients had normal immune function. Conversely, the Japanese study also showed that, in a group of AIDS patients, the number of macrophages, lymphocytes, and giant cells was low. Many organisms were scattered in the lungs. We speculate that intact granuloma cannot form and numerous *Cryptococcus* reproduce in immunocompromised patients, which destroy the structure of the lung and cause the dissemination of pathogens. In our CT images, cavitation within nodules/masses was common in the low CD4+ T cell group. The halo sign was used to describe the hemorrhagic nodules in the CT image, which is fairly common in patients with invasive aspergillosis. A previous study showed that the pathology of the halo sign indicates granulomatous inflammation [14, 29]. In this way, these imaging findings are more likely to appear in the CD4+ T cell normal patients.

Mortality was markedly higher in the patients with low CD4+ T cell counts (28.9%) than in the patients with normal CD4+ T cell counts (5.7%). It may be that the former were likely to suffer from disseminated cryptococcosis. In addition to the site of infection, other reasons were analyzed as follows: First, the lack of effective means of screening in primary hospitals may cause delayed or incorrect diagnoses. Second, in consideration of renal toxicity, we only used azole antifungal agents rather than amphotericin B in the kidney transplant patients in the early period of each case. At the same time, the lack of the amphotericin B liposome is another important reason.

In the current study, patients with low CD4 + T-lymphocyte counts showed more severe clinical symptoms and CT manifestations than those with normal counts. Their Apache II scores were higher than those of the normal CD4 + T lymphocyte patients. In this way, the CD4 + T count can indicate the severity of cryptococcosis and facilitate the estimation of prognosis.

The present study has some limitations. First, it was a retrospective study and examined a small number of patients with cryptococcosis. Second, the abovementioned CD4 + T count indicated a point of the disease and lack of a dynamic evolution process. According to the current study, we should routinely check CD4 + T cell counts and perform a chest CT scan when the patient is highly suspected to have cryptococcosis.

## Conclusions

CD4^+^ T lymphocytes have a significant impact on the clinical and radiological characteristics of pulmonary cryptococcosis. The patients with normal CD4^+^ T cell counts were found to have less fever symptoms and more nodule-like radiographic changes.

## Abbreviations

ATG: rabbit anti-thymocyte globulin
CSF: cerebrospinal fluid
CT: computed tomography
CTD: connective tissue disease
SOT: solid organ transplant
